# Towards Integrated Mid-Infrared Gas Sensors

**DOI:** 10.3390/s19092076

**Published:** 2019-05-04

**Authors:** Daniel Popa, Florin Udrea

**Affiliations:** 1 Department of Engineering, University of Cambridge, Cambridge CB3 0FA, UK; fu10000@cam.ac.uk; 2 ams Sensors UK Limited, Cambridge CB4 0DL, UK

**Keywords:** gas sensors, mid-infrared, integrated sensors, MEMS, CMOS

## Abstract

Optical gas sensors play an increasingly important role in many applications. Sensing techniques based on mid-infrared absorption spectroscopy offer excellent stability, selectivity and sensitivity, for numerous possibilities expected for sensors integrated into mobile and wearable devices. Here we review recent progress towards the miniaturization and integration of optical gas sensors, with a focus on low-cost and low-power consumption devices.

## 1. Introduction

**Figure 1:**
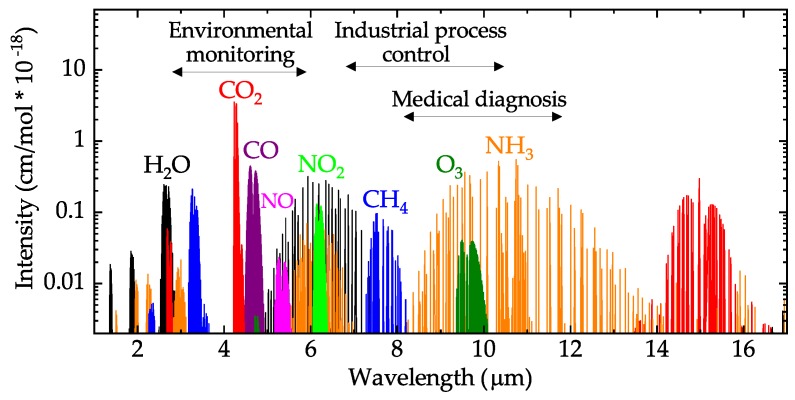
Mid-infrared absorption spectra of selected molecules with their relative intensities. H${}_{2}$O: water; CO${}_{2}$: carbon dioxide; CO: carbon monoxide; NO: nitric oxide; NO${}_{2}$; nitrogen dioxide; CH${}_{4}$: methane; O${}_{3}$: oxygen; NH${}_{3}$: ammonia. Source: HITRAN [10].

Gas sensors are used in a variety of scientific, industrial and commercial applications [1]. Among various sensing techniques [2], sensors based on the interaction of light with gas molecules [3], can offer high sensitivity [4,5], and long-term operation stability [5]. In addition, they have longer lifetimes and shorter response times [3,6], compared to other techniques [2], making them suitable for real-time [7], and in situ [8] detection. Most optical gas sensors rely on absorption spectroscopy [3], where a gas is detected by measuring the light absorbed (due to its interaction with the gas) as a function of wavelength [9]. Many important organic and inorganic molecules [9] have characteristic absorption lines in the mid-infrared (MIR) spectral region (λ∼ 2–20 μm) (Figure 1) [10], corresponding to fundamental vibrational and rotational energy transitions [9]. The MIR fundamental transitions have stronger line strengths than their overtones, typically used in the visible and near-IR regions [9,10]. In addition, spectra are less congested, allowing selective spectroscopic detection of many molecules [9,10]. This molecular “fingerprinting” capability makes MIR gas sensors highly desirable for an increasing number of applications involving chemical analysis, such as industrial process control [11,12,13], environmental monitoring [14,15], and medical diagnosis [16].

With the emerging trend in miniaturization of optical devices based on integration on-chip [17], numerous possibilities are expected for optical gas sensors integrated into smartphones, tablets, wearable and medical devices [18]. Applications such as breath analysis [16,19,20], body tissue and fluid analysis [21], food quality control [22,23,24], identification of impurities or counterfeits [25], or measurement of surfaces, detection of contaminants or identification of solids [26,27] are expected to increase rapidly. Such markets can only be addressed with low-cost, highly reliable and sensitive, and highly compact and stable sensors. In addition, ultra-low power consumption is needed, e.g., to operate such sensors in mobile devices [28,29] or wireless networks [30], power levels below 1 mW are required, at suitably low-costs, less than $2 [18,30,31]. These requirements can be met in integrated optical systems [17,32], by combining miniaturized optical components [33] and waveguides [34] into highly condensed devices. Integration technologies based on complementary metal-oxide semiconductor (CMOS) processes have clear advantages in size, and power [31,35,36], with the possibility of cost reduction leveraging standard high-volume manufacturing for applications such as integration with consumer electronics [31], or sensor networks for the internet of things (IoT) [15,30].

The core of an optical gas sensor is a light source with emission in the range of interest [3]. In addition, dedicated filtering and detection mechanisms are needed [3]. Development of new light sources (e.g., quantum cascade lasers (QCLs) [37,38], light emitting diodes (LEDs) [39,40], and micro-electro-mechanical systems (MEMS)-based thermal emitters [41,42,43]), and detection techniques (e.g., optical [3], and acoustic [44]) have changed the outlook of optical gas sensors over the past two decades. These advancements, in particular the realization of new MIR sources [45], combined with the increasing needs to develop new innovative technologies for healthcare, digital services and other innovation [46], are driving optical gas sensors towards low-cost, mainstream applications [15,19]. Gas sensors based on optical detectors, have been demonstrated for various applications (e.g., breath analysis [19,47], indoor air quality (IAQ) [48], or pollution control [49]). Photoacoustic sensors, using highly-sensitive MEMS microphones, have also emerged as compact, low-cost sensors with high sensitivity and stable operation [44,50,51]. Nevertheless, current optical gas sensing technologies still suffer from drawbacks, e.g., QCLs are expensive complex heterostructures [37,38], LEDs have limited emission for λ > 5 μm [39], and MEMS micro-heaters suffer from poor emissivity [52]. In addition, long (∼cm [3]) optical interaction pathlengths are required to increase the sensor signal response. These limitations motivate research on new materials, novel designs and technologies. Here, we review recent developments towards the miniaturization and integration of optical gas sensors, with a focus on low-cost and low-power consumption devices.

## 2. Optical Gas Sensor Topologies

**Figure 2:**
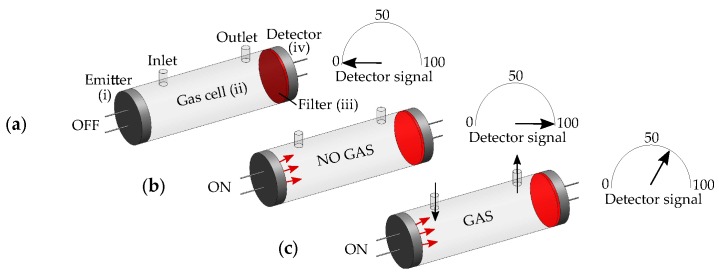
Optical gas sensor based on the Beer–Lambert’s law. (**a**) No signal detected when the emitter is off. (**b**) The detected signal is at a maximum when the emitter is on and no gas is present, and is decreasing (**c**) with the gas concentration, *c*, when the gas is present.

Most optical gas sensors rely on the Beer-Lambert’s law [9], where a gas is detected according to the relation $I\left(\lambda \right)=I_{0}\left(\lambda \right)e^{-\alpha (\lambda )cl}$: where I(λ) and $I_{0}\left(\lambda \right)$ [W/m${}^{2}$] are the detected and emitted optical intensities at the wavelength λ, respectively; α(λ) [L/gm] is the gas absorption coefficient; *c* [g/L] is the gas concentration; and *l* [m] is the light-gas interaction pathlength. A typical sensor (depicted in Figure 2), is comprised of: (i) an emitter to generate $I_{0}\left(\lambda \right)$, (ii) an optical path, *l* (gas cell), to guide light to interact with the gas, (iii) an optical filter to select the range of wavelengths (λ) characteristic to the gas target, and (iv) a detector to detect the absorbed light, I(λ). A common technique relies on nondispersive sensing, where unfiltered light is used to interact with the gas [3,6]. Nondispersive gas sensors allow selective detection (with λ), by filtering the detected light based on the characteristic absorption spectra, α(λ), of the molecular species [9]. Sensors configured with IR emitters and detectors, are traditionally known as nondispersive IR (NDIR) sensors [3], although variations for other spectral regions, or for configurations with acoustic instead of optical detectors [3], share the same operating principle based on the Beer–Lambert’s law [9].

**Figure 3:**
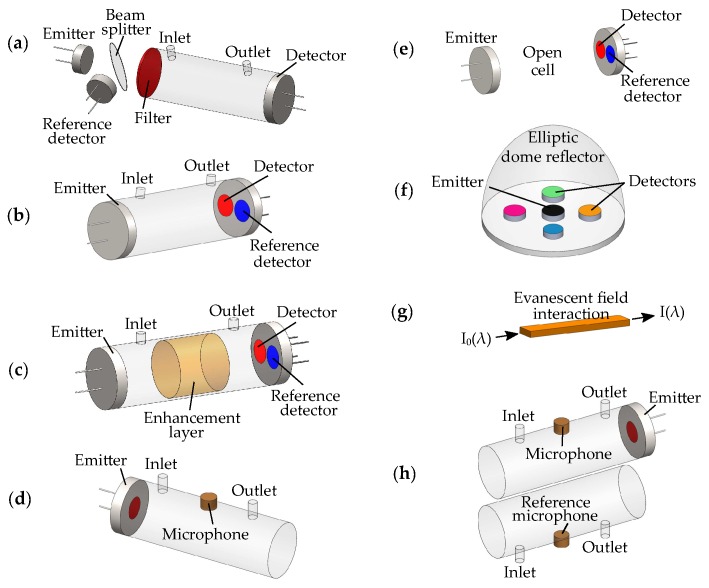
Optical gas sensors topologies. Most commonly used sensors rely on gas cells formed between face-to-face configured emitters and detectors. (**a**) Light can be filtered prior interaction with the gas. An external reference detector can be used to compensate for changes in the emitted light [53]. (**b**) Dual detector configuration, with [48,58] or without [54] filters. (**c**) The gas cell can be reduced by increasing the light-gas interaction, e.g., by using photonic crystals [55], optical cavities [56], multi-pass cells [57], or gas enrichment layers [58]. (**d**) Photoacoustic cell. Acoustic waves created by light-gas interaction are detected by a microphone. It can be resonant [67] or non-resonant [68]. (**e**) Open cell configuration, using either dual optical detection [64] or microphones sealed with target gases [69]. (**f**) Cell with emitter and detector in planar configuration. Multiple optical detectors with filters in the range of interest can be used [59], or microphones sealed with target gases [44]. (**g**) Waveguide sensors based on evanescent field interaction [60,61,62] require optical coupling with both emitters and detectors. (**h**) Dual photoacoustic cell with reference microphone [70,71,72].

Various topologies have been implemented to fabricate optical gas sensors (Figure 3), with the most commonly used based on gas cells formed between face-to-face configured emitters and optical detectors [48,53,54] (Figure 3a–c,e). Strategies to miniaturize the gas cell include: the use of enhancement layers, such as photonic crystals [55], optical cavities [56], multi-pass cells [57], or gas enrichment layers [58], to increase the light-gas interaction (Figure 3c); planar configurations of emitters and detectors [44,59] (Figure 3f); or use of waveguides for evanescent-field interaction [60,61,62] (Figure 3g). The absorbed light, ∼I(λ), is typically detected via an optical detector such as a photodiode [47], thermopile [63], or pyroelectric [64], or an acoustic detector such as a microphone [44]. The sensor response signal, ∼I(λ), is typically extracted by means of a lock-in detection technique, from a known frequency used to modulate the emitter [19,65]. A reference detector is often used to compensate for changes in the emitted light [40,48,53,54,58,59,64]. Additional sensors can be used to compensate for environmental parameters such as temperature, pressure or humidity [44,51,59,63,66]. In this section, we review the performance of current topologies with a focus on miniaturized devices, based on both acoustic and optical detection.

### 2.1. Optical Detection

Gas sensors using optical detectors, to measure I(λ), have been successfully implemented for a variety of gases: acetone (C${}_{3}$H${}_{6}$O) [63], ammonia (NH${}_{3}$) [63], carbon dioxide (CO${}_{2}$) [19,40,43,47,54,58,59,61,64,73], formaldehyde (CH${}_{2}$O) [48], nitric oxide (NO) [53], carbon monoxide (CO) [59,64,74], methane (CH${}_{4}$) [56,59,60,62,64,75,76], or methanol (CH${}_{3}$OH) [77]. Various strategies have been implemented to fabricate gas sensors based on optical detection, Table 1 (Figure 3a–c,e–g). These include designs based on tube-like gas cells formed between face-to-face configured emitters and detectors [19,43,48,53,54,58,63,75,76,77] (Figure 3a–c,e), dome-like gas cells with planar configured emitters and detectors [47,59] (Figure 3f), open cells [40,64,73] (Figure 3e), cavity-enhanced cells [56], or waveguides based on evanescent-field interaction [60,61,62] (Figure 3g). Different light sources have been used such as MEMS heaters [19,43,54,63,64,73,77], LEDs [40,47,48,53,76], distributed feedback lasers (DFBs) [62,75], or QCLs [61], and detectors such as photodiodes [47,48,53,62,76], thermopiles [19,43,54,63], pyroelectric detectors [59,64,77], or photoconductive detectors [56,60,61]. Thus far the most popular configuration is based on face-to-face configured tube-like cells for CO${}_{2}$ detection [19,56,60]. The performance of gas sensors based on optical detection has steadily improved. Table 1 summarizes representative output performances. For example, sensitivities down to tens ppm [19,47,56,63,73] with power consumption possibly below 10 mW [47,73] or even less are now possible in compact low-cost formats [19,47]. Although at the expense of a Helium–Neon (HeNe) laser, ref. [56] presents a remarkably ∼25 μm small MEMS optical cavity for CH${}_{4}$ detection. Note that sensors based on waveguides [60,61,62,78], require external light sources and detectors and suffer from relatively low sensitivities compared to other topologies.

### 2.2. Acoustic Detection

Photoacoustic (PA) gas sensors are also based on the Beer–Lambert’s law [9], where the gas sample is excited by a light source, however, unlike sensors based on optical detection, the response signal I(λ) (proportional to a pressure wave created by the light-gas interaction), is captured by means of acoustic detection [3,79]. Because of their simplicity, and highly reliable performance, PA gas sensors are widely used. They have been implemented for a variety of gases, including: CO${}_{2}$ [15,51,68,69,80,81], CH${}_{4}$ [44,68,69,70,71,81], acetylene (C${}_{2}$H${}_{2}$) [68,82], ethane (C${}_{2}$H${}_{6}$) [68], CO [68,83], ethylene (C${}_{2}$H${}_{4}$) [68], C${}_{3}$H${}_{6}$O [67] sulfur dioxide (SO${}_{2}$) [67], NO [84], hexane (C${}_{6}$H${}_{14}$) [7], oxygen (O${}_{2}$) [72], water (H${}_{2}$O) [81], and nitrogen dioxide (NO${}_{2}$) [66]. Sensitive methods down to few ppb trace gas detection have been reported [7,66]. Various designs have been proposed, based on both resonant (R) (i.e., by tuning the emitter modulation frequency to an acoustic resonance of the cell, thus amplifying the sound signal) [7,66,67,70,71,72,81], and non-resonant (NR) cells [15,44,50,51,69,80,82,84], with light sources including LEDs [15,44,50,67,72], MEMS heaters [51,69,80], QCLs [84], interband cascade lasers (ICLs) [7,71], or DFBs [81,83]. Various strategies have been used to implement the acoustic detector, Table 2. These include designs based on gas-filled [15,44,50,51,69,80,85], or unfilled MEMS microphones [66,67,71,72], optical microphones based on Fabry–Pérot interferometers (FPIs) [68,82], or quartz tuning forks (QTFs) [83,84]. Table 2 summarizes representative operation performances. For example, sensors based on FPIs [68,82] or QTFs [84] feature higher sensitivities. However, these also require more expensive light sources [84], and have larger form factors [68,82,84]. Reference [68] presents a sensor based on a thermal emitter able to detect 6 different gases (C${}_{2}$H${}_{2}$, CH${}_{4}$, C${}_{2}$H${}_{6}$, C${}_{2}$H${}_{4}$, CO and CO${}_{2}$) in the ∼3 to 10 μm range, with remarkably small (sub-ppm) detection limits.

## 3. Path to Miniaturization and Integration

Optical gas sensors provide excellent stability, selectivity, and sensitivity [3,6], being among the most reliable methods for measuring CO${}_{2}$ levels in exhale human breath [16,19,20], and therefore are well suited for next generation medical and consumer electronics end-use applications. However, integration technologies that are efficient, are low-cost and can enable low-power consumption, remain the central challenges of applied modern MIR technologies [45,86]. Although significant effort is being dedicated towards the miniaturization of MIR devices [15,47], progress towards chip-scale, low-cost formats, most needed in a variety of applications, is still in its infancy [17,32]. In this section, we review current progress towards the miniaturization and integration of optical gas sensors, and discuss current major challenges.

### 3.1. MIR Emitters

**Figure 4:**
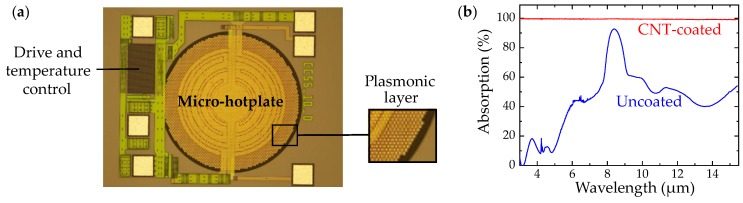
(**a**) Complementary metal-oxide semiconductor (CMOS) integrated plasmonic microhotplate with drive and temperature control. (**b**) Mid-infrared spectra of carbon nanotubes (CNT)-coated and uncoated devices [94].

The high-cost and limited tuning range as well as high-power consumption of current MIR sources [45] (the core of an optical gas sensor), make the use of optical gas sensors with low-cost, battery-operated systems an ongoing problem, and even more so with wireless systems [15,30]. For example, despite the success of QCLs in the MIR [37,38], their high-cost (∼$1000) and high-power consumption have limited their application to consumer electronics. MIR LEDs can offer lower power consumption with overall high efficiencies [39,40], however, their operation above ∼5 μm is challenging [45] and comes at significantly increased costs (∼$100). Nevertheless, renewed scientific interest in the miniaturization of low-cost optical gas sensors [43,54,63], is being fueled by advances in silicon micromachining [36,87]. Recently, membrane microhotplates based on MEMS technology [88,89,90] (Figure 4), came up as compact, integrated thermal light sources [42,43,91]. MEMS heaters are proven to be energy efficient [90], allow for rapid modulation owing to their low thermal mass [19,90], and are compatible with standard CMOS foundry processes [19,90]. They are typically used with CMOS compatible thermal detectors (e.g., thermopiles [19,43,54,63,92], bolometers [73], or pyroelectric detectors [59,64,77]), as they allow broadband MIR detection at room temperature [93] with minimum manufacturing costs [36]. However, standard CMOS materials exhibit inherently low MIR emissivity/absorptivity, especially for wavelengths <8 μm, which makes additional post-CMOS/MEMS blackening layers and filter elements necessary [36], often needed to fulfil applications such as spectroscopy.

We have developed various CMOS microhotplates based on tungsten metallization, as well as several thermal engineering techniques to enhance and tailor their MIR proprieties, Figure 4. Tungsten is an interconnect metal found in high temperature CMOS processes, and can enable stable MIR emitters [90] with excellent device reproducibility and the possibility of a wide range of on-chip circuitry, at very low cost [36]. We have engineered highly efficient plasmonic metal structures to enhance the microhotplate MIR emission via excitation of surface plasmon resonances [43], which can be broadly tuned by varying the structure unit cell geometry. CMOS integrated MIR emitters, with drive and temperature control, can feature membrane diameters as small as 600 μm, and have ∼50 mW DC power consumption (∼1 mW optical output power), when operated at 550 ${}^{\circ }$C, with good emission for λ > 8 μm (Figure 4a). We have also showed that the radiation properties of carbon nanotubes (CNTs) can significantly enhance both emissivity [95] and absorptivity [94] of MIR devices, due to their blackbody-like behaviour (nearly unity) (Figure 4b).

### 3.2. Spectroscopy

One of the most important area of research in MIR technologies is to develop compact and affordable spectroscopic devices [17,45]. This would give immediate access to a broad range of applications while at the same time supporting developments in new areas [18]. Currently, on-line, real-time spectroscopic gas sensors, are used for the detection of single analytes at trace levels, or two to three species at most at the same time [59,63,64,77]. Main limitations include the high-cost and limited tuning ranges of MIR sources [45]. Proposed solutions for miniaturization of MIR spectrometers include linear variable optical filters [96], interferometer arrays [97], Fabry–Pérot interferometers (FPIs) [77,98], or MEMS Fourier transform IR (FTIR)-based spectrometers [99]. However, these require high-power lasers [96,97], have limited tuning ranges [77,96,97,99], or moving parts [77,99] and have relatively high costs [77]. Nondispersive gas sensors relax the requirements on the MIR light sources and detectors [3,19,73], hence exploiting standard CMOS processes is an attractive route towards the fabrication of low-cost integrated thermal emitters and detectors [36]. For this reason, membrane MEMS devices emerged as MIR light sources [36,89,90,91] and detectors [92,100,101], with various thermal engineering techniques, e.g., based on: photonic crystals [55], multi-quantum well structures [102], resonant-cavities [103], carbon nanoparticle adlayers [94,95], and plasmonic metamaterials [42,43]. Among these, the overall broadband emission enhancement (almost unity) offered by carbon-based nanomaterial adlayers [94,95], are of particular interest for spectroscopy. Plasmonic/metamaterial concepts [42,43] can be successfully applied to nondispersive sensors [54], based on both MEMS thermal emitters [43,63] and detectors [54], to enable wavelength tailored single- and multi-band [104], as well as polarization- and angle-independent [105] operation. In absence of these optical engineering approaches, CMOS MIR thermal devices have shown poor/non-optimal spectral performance exclusively defined by the used material proprieties [92,94,95].

**Figure 5:**
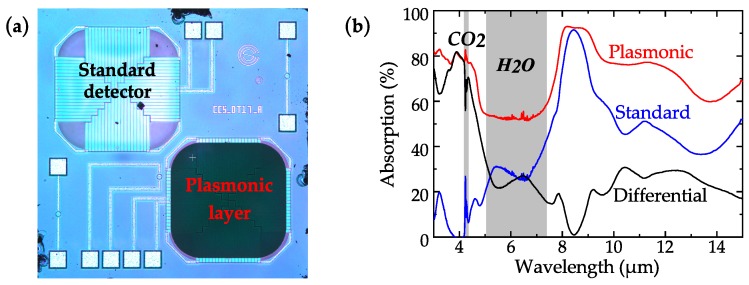
(**a**) Differential filter-free detection, and (**b**) spectral response. Adapted from ref. [54].

We have developed a filter-free technique for the detection of CO${}_{2}$ based on CMOS plasmonic emitters and detectors [54] (Figure 5), that could be applied for spectroscopic detection across the entire MIR spectrum. The detector signal is computed differentially between the plasmonic and non-plasmonic cells as shown in Figure 5b. Note that except for the plasmonic layer, the two integrated detector cells are identical. The differential signal has a peak around 4.26 μm in the CO${}_{2}$ detection range and low absorptivity at other wavelengths. More recently, arrays of wavelength-dependent detectors, based on similar plasmonic/metamaterial thermal engineering concepts have been proposed [106,107,108], to enable spectroscopic detection across various bands in the MIR.

### 3.3. Acoustic vs. Optic Detection

Compared to traditional nondispersive gas sensors, PA gas sensors present several advantages. They do not require optical detectors and are wavelength independent. The absorbed light, ∼I(λ)∼c, is measured directly (i.e., not relative to a background), meaning PA is highly accurate, with very little instability [3,79]. Other advantages include smaller (sub-cm [51,69,80]) optical pathlengths, *l*, and more robust setups [3,44]. Among these, non-resonant PA gas sensors are more stable, feature lower modulating frequencies and require smaller volumes and pathlengths, hence are less susceptible to noise [15,44,50,51,69,80,82,84]. Resonant PA sensors can, however, offer higher sensitivities, but their stability is affected by environmental parameters, such as temperature and pressure [7,66,67,70,71,72,81]. Despite these benefits, only recently efforts towards non-resonant PA gas sensor miniaturization have been reported, e.g., based on thermal emitters [51,69,80], and LEDs [15,44,50] in combination with microphones, or LEDs and QTFs [109]. Among these, sensors based on highly-sensitive MEMS microphones (employed to detect pressure pulses modulated at audio frequencies) are easy to integrate [110], and offer sensitivities down to ∼tens ppm [15,67,80], with overall small power consumption [50], and form factor [44,50]. PA is unique since it is a direct monitor of a sample nonradiative relaxation channels and, hence, complements absorption spectroscopic techniques [9]. Although PA spectra can be recorded by measuring the sound at different wavelengths of light [79], it requires tunable or multiple MIR sources centred at specific wavelengths, which are not available at low-cost and/or low-power consumption [45]. On the other hand, spectroscopic detection techniques based on arrays of plasmonic detectors [54,106,107,108] can be achieved at very low cost and minimum power consumption.

### 3.4. Electronics and Signal Processing

**Figure 6:**
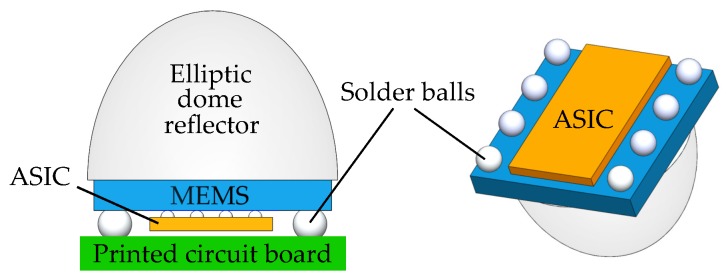
Integrated optical gas sensor concept. Planar integration of emitters and detectors within the same micro-electro-mechanical systems (MEMS) chip, facing an optimized multi-reflection elliptic mirror (gas cell). The sensor signal can be enhanced with an on-chip amplifier and have the first analogue processing level done on-chip with more complex signal processing done externally through the use of an application-specific integrated circuit (ASIC). The MEMS and ASIC chips are attached via face-to-face flip-chip bonding.

Optical gas sensors require signal amplification and processing techniques to increase their signal-to-noise ratio. A common technique relies on lock-in amplification [19,65], where the sensor response signal is recovered from noise by extracting it at a specific reference frequency, typically used to modulate the emitter (e.g., light pulses). Bench-top lock-in amplifiers are widely used in optical gas sensor setups [60,61,66,68,70,71,81,82,84], however, they are not suitable for use with portable sensing devices, and even less so with integrated circuits (ICs). Miniaturized, IC-based lock-in amplifiers can be used to implement optical gas sensors, with relatively good performance [19]. Other techniques include the use of digital signal processors (DSPs) based on microcontrollers (MCUs) [43,75,80] or field programmable gate arrays (FPGAs) [7]. MCU-implemented digital lock-in amplifiers [43,75,80], or fast Fourier transform (FFT)-based techniques [44,69], are increasingly used. An integrated optical gas sensor concept is shown in Figure 6.

## 4. Outlook

Optical gas sensors based on MIR absorption spectroscopy offer excellent stability, selectivity, and sensitivity for a growing number of potential applications. Experimental setups that combine low-cost and low-power consumption emitters and detectors, are attractive prospects for battery-operated mobile devices and networks. CMOS-based technologies increase device fabrication flexibility, in addition to having economic advantages. Among various configurations, sensors based on dome-like gas cells [44,47,50,59], allow for planar integration of emitters and detectors, and could be used to further reduce their size. The main challenge is the relatively small pathlengths, *l*, which can be addressed by further device optimization, e.g., based on multi-pass designs [57,74,111], or use of photonic crystals [55,112] or optical cavities [56] for enhanced absorption. More recently, photoacoustic gas sensors, based on low-cost commercially available MEMS microphones have emerged as simple, compact, and highly reliable devices [15,50].

Most current MIR light sources are expensive and suffer from high-power consumption [45]. MEMS microhotplates have clear advantages in terms of costs, and could potentially (given their full CMOS compatibility) enable sensors with costs below $1. In addition, they have been demonstrated at various wavelengths in the MIR [43,54], with power-consumption possibly below 1 mW. In principle, optical gas sensors based on plasmonic microhotplates could operate across the entire MIR range with relatively high performance [68]. The integration of nanostructures and nanomaterials in MEMS silicon technology could, in principle, produce novel broadband MIR tunable sources. The recent demonstration of a filter-free gas sensor shows the possibility of using this approach for a broad spectral range [54]. These integration technologies could be applied to various gas sensor designs, based on both optical and acoustic detection.

## Figures and Tables

**Table 1:** Gas sensors based on optical detection. λ: operating wavelength; *l*: optical path; MEMS: micro-electro-mechanical systems; FF: face-to-face; //: unreported; LED: light emitting diode; DFB: distributed feedback laser; QCL: quantum cascade laser; MCT: mercury cadmium telluride; WG: waveguide; C${}_{3}$H${}_{6}$O: acetone; NH${}_{3}$: ammonia; CO${}_{2}$: carbon dioxide; CH${}_{2}$O: formaldehyde; NO: nitric oxide; CO: carbon monoxide; CH${}_{4}$: methane; C${}_{2}$H${}_{4}$O${}_{2}$: methyl formate; CH${}_{3}$OH: methanol.

Emitter	Detector	λ [μm]	*l* [cm]	Gas Detected	Detection Limit [ppm]	Topology (cell)	Power Consumption [mW]	Ref.
MEMS	Thermopile	8.26	10	C${}_{3}$H${}_{6}$O	50	FF	//	
heater		10.6		NH${}_{3}$	10			[63]
LED	Photodiode	4.26	//	CO${}_{2}$	//	Planar	35	
						(dome)		[47]
MEMS	Bolometer	4.26	8	CO${}_{2}$	30	Planar	45	
heater						(open)		[73]
LED	Photodiode	0.34	19.5	CH${}_{2}$O	4.3	FF	//	[48]
LED	Photodiode	0.226	50	NO	2	FF	//	[53]
DFB	//	1.65	20	CH${}_{4}$	11	FF	//	[75]
MEMS	Pyroelectric	4.65	39.3	CO	8.8	Planar	//	
heater		4.26		CO${}_{2}$	8.7	(open)		
		3.31		CH${}_{4}$	10.3			[64]
MEMS	Thermopile	4.26	8	CO${}_{2}$	50	FF	350	
heater								[19]
//	//	4.26	0.5	CO${}_{2}$	∼400	FF	//	[58]
HeNe	PbSe	3.4	0.15	CH${}_{4}$	//	Cavity	//	
laser						(∼25 μm)		[56]
Lamp	Pyroelectric	4.66	2.6	CO	//	Planar	//	
		4.26		CO${}_{2}$		(dome)		
		3.33		CH${}_{4}$				[59]
MEMS	Thermopile	4.26	7.5	CO${}_{2}$	//	FF	∼80	
heater								[43]
AlInSb	InSb	4.2	8	CO${}_{2}$	1000	Planar	//	
LED						(open)		[40]
MEMS	Thermopile	4.26	7	CO${}_{2}$	//	FF	∼50	
heater								[54]
LED	Photodiode	1.66	12	CH${}_{4}$	100	FF	//	
	(InGaAs)							[76]
MEMS	Pyroelectric	8.4	30	C${}_{2}$H${}_{4}$O${}_{2}$	165	FF	//	
heater		9.6		CH${}_{3}$OH	184			[77]
Laser	PbTe	3.31	0.5	CH${}_{4}$	1000	WG	//	[60]
QCL	MCT	4.23	1	CO${}_{2}$	5000	WG	//	[61]
DFB	InGaAs	1.65	10	CH${}_{4}$	100	WG	//	[62]

**Table 2:** Gas sensors based on acoustic detection. F-MEMS: gas filled-MEMS; FPI: Fabry–Pérot interferometer; ICLED: interband cascade light emitting device; QTF: quartz tuning fork; ICL: interband cascade laser; NR: non-resonant; R: resonant; //: unreported; CO${}_{2}$: carbon dioxide; CH${}_{4}$: methane; C${}_{2}$H${}_{2}$: acetylene; C${}_{2}$H${}_{6}$: ethane; CO: carbon monoxide; C${}_{2}$H${}_{4}$: ethylene; C${}_{3}$H${}_{6}$O: acetone; SO${}_{2}$: sulfur dioxide; NO: nitric oxide; C${}_{6}$H${}_{14}$: hexane; O${}_{2}$: oxygen; H${}_{2}$O: water; NO${}_{2}$: nitrogen dioxide.

Emitter	Detector	λ [μm]	*l* [cm]	Gas Detected	Detection Limit [ppm]	Cell	Power Consumption [mW]	Ref.
LED	F-MEMS	4.2	3	CO${}_{2}$	50	NR	//	
	microphone							[15]
LED	F-MEMS	3.4	1.2	CH${}_{4}$	2500	NR	//	
	microphone							[44]
MEMS	F-MEMS		0.44	CO${}_{2}$	//	NR	//	
heater	microphone		0.64	CH${}_{4}$				[69]
LED	F-MEMS	4.3	1.2	CO${}_{2}$	100	NR	48	
	microphone							[50]
Thermal	Diaphragm	3.05	//	C${}_{2}$H${}_{2}$	0.11	NR	//	
emitter	FPI	3.22		CH${}_{4}$	0.21			
		3.37		C${}_{2}$H${}_{6}$	0.13			
		4.26		CO${}_{2}$	0.48			
		4.68		CO	0.15			
		10.68		C${}_{2}$H${}_{4}$	0.16			[68]
LED	MEMS	0.285	15	C${}_{3}$H${}_{6}$O	40	R	//	
	microphone			SO${}_{2}$	2			[67]
Laser	Cantilever	1.53	2	C${}_{2}$H${}_{2}$	0.015	NR	//	
	FPI							[82]
ICLED	Microphone	3.2	11	CH${}_{4}$	3.6	R	//	[70]
QCL	QTF	5.26	19.3	NO	0.004	Cavity	//	[84]
LED	MEMS	0.76	//	O${}_{2}$	//	R	//	
	microphone							[72]
MEMS	F-MEMS		0.38	CO${}_{2}$	200	NR	//	
heater	microphone							[51]
MEMS	F-MEMS		0.5	CO${}_{2}$	50	NR	//	
heater	microphone							[80]
ICL	MEMS	3.36	//	CH${}_{4}$	0.32	R	//	
	microphone							[71]
ICL	Microphone	3.38	∼1	C${}_{6}$H${}_{14}$	0.4*10${}^{-3}$	R	//	[7]
DFB	Microphone	2	12	CO${}_{2}$	12	R	//	
		1.6	11	CH${}_{4}$	0.2			
		1.4	10	H${}_{2}$O	0.1			[81]
DFB	QTF	2.33	∼3	CO	0.021	NR	4200	[83]
DFB	MEMS	1.5	3	C${}_{2}$H${}_{2}$	0.03	NR	//	
	microphone							[57]
Laser	MEMS	0.450	38.7	NO${}_{2}$	33*10${}^{-6}$	R	//	
	microphone							[66]

## References

[1] Potyrailo R.A. (2016). Multivariable Sensors for Ubiquitous Monitoring of Gases in the Era of Internet of Things and Industrial Internet. Chem. Rev..

[2] Liu X., Cheng S., Liu H., Hu S., Zhang D., Ning H. (2012). A Survey on Gas Sensing Technology. Sensors.

[3] Hodgkinson J., Tatam R.P. (2013). Optical gas sensing: A review. Meas. Sci. Technol..

[4] Galli I., Bartalini S., Borri S., Cancio P., Mazzotti D., De Natale P., Giusfredi G. (2011). Molecular Gas Sensing Below Parts Per Trillion: Radiocarbon-Dioxide Optical Detection. Phys. Rev. Lett..

[5] Tomberg T., Vainio M., Hieta T., Halonen L. (2018). Sub-parts-per-trillion level sensitivity in trace gas detection by cantilever-enhanced photo-acoustic spectroscopy. Sci. Rep..

[6] Dinh T.V., Choi I.Y., Son Y.S., Kim J.C. (2016). A review on non-dispersive infrared gas sensors: Improvement of sensor detection limit and interference correction. Sens. Actuators B Chem..

[7] Petersen J.C., Balslev-Harder D., Pelevic N., Brusch A., Persijn S., Lassen M. Flow immune photoacoustic sensor for real-time and fast sampling of trace gases. Proceedings of the Photonic Instrumentation Engineering V.

[8] Dooly G., Clifford J., Leen G., Lewis E. (2012). Mid-infrared point sensor for in situ monitoring of CO_2_ emissions from large-scale engines. Appl. Opt..

[9] Bernath P.F. (2016). Spectra of Atoms and Molecules.

[10] Gordon I., Rothman L., Hill C., Kochanov R., Tan Y., Bernath P., Birk M., Boudon V., Campargue A., Chance K. (2017). The HITRAN2016 molecular spectroscopic database. J. Quant. Spectrosc. Radiat. Transf..

[11] Schmid T. (2006). Photoacoustic spectroscopy for process analysis. Anal. Bioanal. Chem..

[12] Goldenstein C.S., Spearrin R.M., Jeffries J.B., Hanson R.K. (2017). Infrared laser-absorption sensing for combustion gases. Prog. Energy Combust. Sci..

[13] Willer U., Saraji M., Khorsandi A., Geiser P., Schade W. (2006). Near- and mid-infrared laser monitoring of industrial processes, environment and security applications. Opt. Lasers Eng..

[14] Schieweck A., Uhde E., Salthammer T., Salthammer L.C., Morawska L., Mazaheri M., Kumar P. (2018). Smart homes and the control of indoor air quality. Renew. Sustain. Energy Rev..

[15] Ortiz Perez A., Bierer B., Scholz L., Wöllenstein J., Palzer S. (2018). A Wireless Gas Sensor Network to Monitor Indoor Environmental Quality in Schools. Sensors.

[16] Metsälä M. (2018). Optical techniques for breath analysis: From single to multi-species detection. J. Breath Res..

[17] Sieger M., Mizaikoff B. (2016). Toward On-Chip Mid-Infrared Sensors. Anal. Chem..

[18] Bogue R. (2013). Recent developments in MEMS sensors: A review of applications, markets and technologies. Sens. Rev..

[19] Vincent T.A., Gardner J.W. (2016). A low cost MEMS based NDIR system for the monitoring of carbon dioxide in breath analysis at ppm levels. Sens. Actuators B Chem..

[20] Henderson B., Khodabakhsh A., Metsälä M., Ventrillard I., Schmidt F.M., Romanini D., Ritchie G.A.D., te Lintel Hekkert S., Briot R., Risby T. (2018). Laser spectroscopy for breath analysis: Towards clinical implementation. Appl. Phys. B.

[21] Takamura A., Watanabe K., Akutsu T., Ozawa T. (2018). Soft and Robust Identification of Body Fluid Using Fourier Transform Infrared Spectroscopy and Chemometric Strategies for Forensic Analysis. Sci. Rep..

[22] Loutfi A., Coradeschi S., Mani G.K., Shankar P., Rayappan J.B.B. (2015). Electronic noses for food quality: A review. J. Food Eng..

[23] Clara D., Pezzei C.K., Schönbichler S.A., Popp M., Krolitzek J., Bonn G.K., Huck C.W. (2016). Comparison of near-infrared diffuse reflectance (NIR) and attenuated-total-reflectance mid-infrared (ATR-IR) spectroscopic determination of the antioxidant capacity of Sambuci flos with classic wet chemical methods (assays). Anal. Methods.

[24] Wilson R.H., Tapp H.S. (1999). Mid-infrared spectroscopy for food analysis: Recent new applications and relevant developments in sample presentation methods. Trends Anal. Chem..

[25] Lawson G., Ogwu J., Tanna S. (2018). Quantitative screening of the pharmaceutical ingredient for the rapid identification of substandard and falsified medicines using reflectance infrared spectroscopy. PLoS ONE.

[26] Hutengs C., Ludwig B., Jung A., Eisele A., Vohland M. (2018). Comparison of Portable and Bench-Top Spectrometers for Mid-Infrared Diffuse Reflectance Measurements of Soils. Sensors.

[27] Soriano-Disla J.M., Janik L.J., Viscarra Rossel R.A., Macdonald L.M., McLaughlin M.J. (2014). The Performance of Visible, Near-, and Mid-Infrared Reflectance Spectroscopy for Prediction of Soil Physical, Chemical, and Biological Properties. Appl. Spectrosc. Rev..

[28] Carroll A., Heiser G. (2010). An Analysis of Power Consumption in a Smartphone. Proceedings of the 2010 USENIX Conference on USENIX Annual Technical Conference, USENIXATC’10.

[29] Mao Y., You C., Zhang J., Huang K., Letaief K.B. (2017). A Survey on Mobile Edge Computing: The Communication Perspective. IEEE Commun. Surv. Tutor..

[30] Palattella M.R., Dohler M., Grieco A., Rizzo G., Torsner J., Engel T., Ladid L. (2016). Internet of Things in the 5G Era: Enablers, Architecture, and Business Models. IEEE J. Sel. Areas Commun..

[31] Gardner J.W., Guha P.K., Udrea F., Covington J.A. (2010). CMOS Interfacing for Integrated Gas Sensors: A Review. IEEE Sens. J..

[32] Kita D.M., Lin H., Agarwal A., Richardson K., Luzinov I., Gu T., Hu J. (2017). On-Chip Infrared Spectroscopic Sensing: Redefining the Benefits of Scaling. IEEE J. Sel. Top. Quantum Electron..

[33] Singh V., Lin P.T., Patel N., Lin H., Li L., Zou Y., Deng F., Ni C., Hu J., Giammarco J. (2014). Mid-infrared materials and devices on a Si platform for optical sensing. Sci. Technol. Adv. Mater..

[34] Testa G., Persichetti G., Bernini R. (2018). Hollow-Core-Integrated Optical Waveguides for Mid-IR Sensors. IEEE J. Sel. Top. Quantum Electron..

[35] Baltes H., Brand O. (2001). CMOS-based microsensors and packaging. Sens. Actuators A Phys..

[36] Udrea F., Luca A.D. CMOS technology platform for ubiquitous microsensors. Proceedings of the 2017 International Semiconductor Conference (CAS).

[37] Vitiello M.S., Scalari G., Williams B., Natale P.D. (2015). Quantum cascade lasers: 20 years of challenges. Opt. Express.

[38] Razeghi M., Lu Q.Y., Bandyopadhyay N., Zhou W., Heydari D., Bai Y., Slivken S. (2015). Quantum cascade lasers: From tool to product. Opt. Express.

[39] Fujita H., Ueno K., Morohara O., Camargo E., Geka H., Shibata Y., Kuze N. (2018). AlInSb Mid-Infrared LEDs of High Luminous Efficiency for Gas Sensors. Phys. Status Solidi (a).

[40] Camargo E.G., Goda Y., Morohara O., Fujita H., Geka H., Ueno K., Shibata Y., Kuze N. NDIR gas sensing using high performance AlInSb mid-infrared LEDs as light source. Proceedings of the SPIE, Infrared Sensors, Devices, and Applications VII.

[41] Luca A.D., Ali S.Z., Udrea F. On the reproducibility of CMOS plasmonic mid-IR thermal emitters. Proceedings of the 2017 International Semiconductor Conference (CAS).

[42] Lochbaum A., Fedoryshyn Y., Dorodnyy A., Koch U., Hafner C., Leuthold J. (2017). On-Chip Narrowband Thermal Emitter for Mid-IR Optical Gas Sensing. ACS Photonics.

[43] Pusch A., De Luca A., Oh S.S., Wuestner S., Roschuk T., Chen Y., Boual S., Ali Z., Phillips C.C., Hong M. (2015). A highly efficient CMOS nanoplasmonic crystal enhanced slow-wave thermal emitter improves infrared gas-sensing devices. Sci. Rep..

[44] Wittstock V., Scholz L., Bierer B., Perez A.O., Wöllenstein J., Palzer S. (2017). Design of a LED-based sensor for monitoring the lower explosion limit of methane. Sens. Actuators B Chem..

[45] Jung D., Bank S., Lee M.L., Wasserman D. (2017). Next-generation mid-infrared sources. J. Opt..

[46] Schork N.J. (2015). Personalized medicine: Time for one-person trials. Nature.

[47] Fleming L., Gibson D., Song S., Li C., Reid S. (2018). Reducing N2O induced cross-talk in a NDIR CO_2_ gas sensor for breath analysis using multilayer thin film optical interference coatings. Surf. Coat. Technol..

[48] Davenport J.J., Hodgkinson J., Saffell J.R., Tatam R.P. (2018). Non-Dispersive Ultra-Violet Spectroscopic Detection of Formaldehyde Gas for Indoor Environments. IEEE Sens. J..

[49] Fanchenko S., Baranov A., Savkin A., Sleptsov V. (2016). LED-based NDIR natural gas analyzer. IOP Conf. Ser. Mater. Sci. Eng..

[50] Scholz L., Perez A.O., Bierer B., Eaksen P., Wöllenstein J., Palzer S. (2017). Miniature Low-Cost Carbon Dioxide Sensor for Mobile Devices. IEEE Sens. J..

[51] Huber J., Weber C., Eberhardt A., Wöllenstein J. (2016). Photoacoustic CO_2_-Sensor for Automotive Applications. Procedia Eng..

[52] Liu X., Tyler T., Starr T., Starr A.F., Jokerst N.M., Padilla W.J. (2011). Taming the Blackbody with Infrared Metamaterials as Selective Thermal Emitters. Phys. Rev. Lett..

[53] Mehnke F., Guttmann M., Enslin J., Kuhn C., Reich C., Jordan J., Kapanke S., Knauer A., Lapeyrade M., Zeimer U. (2017). Gas Sensing of Nitrogen Oxide Utilizing Spectrally Pure Deep UV LEDs. IEEE J. Sel. Top. Quantum Electron..

[54] Luca A.D., Ali S.Z., Hopper R., Boual S., Gardner J.W., Udrea F. Filterless non-dispersive infra-red gas detection: A proof of concept. Proceedings of the 2017 IEEE 30th International Conference on Micro Electro MechanicalSystems (MEMS).

[55] Kraeh C., Martinez-Hurtado J.L., Popescu A., Hedler H., Finley J.J. (2018). Slow light enhanced gas sensing in photonic crystals. Opt. Mater..

[56] Ayerden N.P., de Graaf G., Enoksson P., Wolffenbuttel R.F. A highly miniaturized NDIR methane sensor. Proceedings of the SPIE, Micro-Optics.

[57] Chen K., Zhang B., Liu S., Jin F., Guo M., Chen Y., Yu Q. (2019). Highly sensitive photoacoustic gas sensor based on multiple reflections on the cell wall. Sens. Actuators A Phys..

[58] Moumen S., Raible I., Krauß A., Wöllenstein J. (2016). Infrared investigation of CO_2_ sorption by amine based materials for the development of a NDIR CO_2_ sensor. Sens. Actuators B Chem..

[59] Tan Q., Tang L., Yang M., Xue C., Zhang W., Liu J., Xiong J. (2015). Three-gas detection system with IR optical sensor based on NDIR technology. Opt. Lasers Eng..

[60] Su P., Han Z., Kita D., Becla P., Lin H., Deckoff-Jones S., Richardson K., Kimerling L.C., Hu J., Agarwal A. (2019). Monolithic on-chip mid-IR methane gas sensor with waveguide-integrated detector. Appl. Phys. Lett..

[61] Ranacher C., Consani C., Tortschanoff A., Jannesari R., Bergmeister M., Grille T., Jakoby B. (2018). Mid-infrared absorption gas sensing using a silicon strip waveguide. Sens. Actuators A Phys..

[62] Tombez L., Zhang E.J., Orcutt J.S., Kamlapurkar S., Green W.M.J. (2017). Methane absorption spectroscopy on a silicon photonic chip. Optica.

[63] Xing Y., Urasinska-Wojcik B., Gardner J.W. Plasmonic enhanced CMOS non-dispersive infrared gas sensor for acetone and ammonia detection. Proceedings of the 2018 IEEE International Instrumentation and Measurement Technology Conference (I2MTC).

[64] Dong M., Zheng C., Miao S., Zhang Y., Du Q., Wang Y., Tittel F.K. (2017). Development and Measurements of a Mid-Infrared Multi-Gas Sensor System for CO, CO_2_ and CH_4_ Detection. Sensors.

[65] Sklorz A., Janßen S., Lang W. (2012). Detection limit improvement for NDIR ethylene gas detectors using passive approaches. Sens. Actuators B Chem..

[66] Rück T., Bierl R., Matysik F.M. (2017). Low-cost photoacoustic NO_2_ trace gas monitoring at the pptV-level. Sens. Actuators A Phys..

[67] El-Safoury M., Weber C., Schmitt K., Pernau H., Willing B., Woellenstein J. Photoacoustic gas detector for the monitoring of sulfur dioxide content in ship emissions. Proceedings of the 19th ITG/GMA-Symposium Sensors and Measuring Systems.

[68] Gong Z., Chen K., Yang Y., Zhou X., Yu Q. (2018). Photoacoustic spectroscopy based multi-gas detection using high-sensitivity fiber-optic low-frequency acoustic sensor. Sens. Actuators B Chem..

[69] Knobelspies S., Bierer B., Ortiz Perez A., Wöllenstein J., Kneer J., Palzer S. (2016). Low-cost gas sensing system for the reliable and precise measurement of methane, carbon dioxide and hydrogen sulfide in natural gas and biomethane. Sens. Actuators B Chem..

[70] Zheng H., Lou M., Dong L., Wu H., Ye W., Yin X., Kim C.S., Kim M., Bewley W.W., Merritt C.D. (2017). Compact photoacoustic module for methane detection incorporating interband cascade light emitting device. Opt. Express.

[71] Rouxel J., Coutard J.G., Gidon S., Lartigue O., Nicoletti S., Parvitte B., Vallon R., Zéninari V., Glière A. (2015). Development of a Miniaturized Differential Photoacoustic Gas Sensor. Procedia Eng..

[72] Avramescu V., Gologanu M. Oxygen sensor based on photo acoustic effect. Proceedings of the 2017 International Semiconductor Conference (CAS).

[73] Barritault P., Brun M., Lartigue O., Willemin J., Ouvrier-Buffet J.L., Pocas S., Nicoletti S. (2013). Low power CO_2_ NDIR sensing using a micro-bolometer detector and a micro-hotplate IR-source. Sens. Actuators B Chem..

[74] Ghorbani R., Schmidt F.M. (2017). ICL-based TDLAS sensor for real-time breath gas analysis of carbon monoxide isotopes. Opt. Express.

[75] Zheng C.T., Huang J.Q., Ye W.L., Lv M., Dang J.M., Cao T.S., Chen C., Wang Y.D. (2013). Demonstration of a portable near-infrared CH4 detection sensor based on tunable diode laser absorption spectroscopy. Infrared Phys. Technol..

[76] Massie C., Stewart G., McGregor G., Gilchrist J.R. (2006). Design of a portable optical sensor for methane gas detection. Sens. Actuators B Chem..

[77] Genner A., Gasser C., Moser H., Ofner J., Schreiber J., Lendl B. (2017). On-line monitoring of methanol and methyl formate in the exhaust gas of an industrial formaldehyde production plant by a mid-IR gas sensor based on tunable Fabry-Pérot filter technology. Anal. Bioanal. Chem..

[78] Han Z., Lin P., Singh V., Kimerling L., Hu J., Richardson K., Agarwal A., Tan D.T.H. (2016). On-chip mid-infrared gas detection using chalcogenide glass waveguide. Appl. Phys. Lett..

[79] Bozóki Z., Pogány A., Szabó G. (2011). Photoacoustic Instruments for Practical Applications: Present, Potentials, and Future Challenges. Appl. Spectrosc. Rev..

[80] Ambs A., Huber J., Wöllenstein J. Compact Photoacoustic Gas Measuring System for Carbon Dioxide Indoor Monitoring Applications. Proceedings of the AMA Conferences.

[81] Liu K., Mei J., Zhang W., Chen W., Gao X. (2017). Multi-resonator photoacoustic spectroscopy. Sens. Actuators B Chem..

[82] Zhou S., Slaman M., Iannuzzi D. (2017). Demonstration of a highly sensitive photoacoustic spectrometer based on a miniaturized all-optical detecting sensor. Opt. Express.

[83] He Y., Ma Y., Tong Y., Yu X., Tittel F.K. (2019). A portable gas sensor for sensitive CO detection based on quartz-enhanced photoacoustic spectroscopy. Opt. Laser Technol..

[84] Wojtas J., Gluszek A., Hudzikowski A., Tittel F. (2017). Mid-Infrared Trace Gas Sensor Technology Based on Intracavity Quartz-Enhanced Photoacoustic Spectroscopy. Sensors.

[85] Scholz L., Palzer S. (2016). Photoacoustic-based detector for infrared laser spectroscopy. Appl. Phys. Lett..

[86] Soref R. (2010). Mid-infrared photonics in silicon and germanium. Nat. Photonics.

[87] Lammel G. The future of MEMS sensors in our connected world. Proceedings of the 2015 28th IEEE International Conference on Micro Electro Mechanical Systems (MEMS).

[88] Udrea F., Gardner J.W., Setiadi D., Covington J.A., Dogaru T., Lu C.C., Milne W.I. (2001). Design and simulations of SOI CMOS micro-hotplate gas sensors. Sens. Actuators B Chem..

[89] Barritault P., Brun M., Gidon S., Nicoletti S. (2011). Mid-IR source based on a free-standing microhotplate for autonomous CO_2_ sensing in indoor applications. Sens. Actuators A Phys..

[90] Ali S.Z., De Luca A., Hopper R., Boual S., Gardner J., Udrea F. (2015). A Low-Power, Low-Cost Infra-Red Emitter in CMOS Technology. IEEE Sens. J..

[91] Pühringer G., Jakoby B. (2017). Efficient Vertical-Cavity Mid-IR Thermal Radiation to Silicon-Slab Waveguide Coupling Using a Shallow Blazed Grating. Proceedings.

[92] Hopper R., Ali S., Chowdhury M., Boual S., De Luca A., Gardner J.W., Udrea F. (2014). A CMOS-MEMS Thermopile with an Integrated Temperature Sensing Diode for Mid-IR Thermometry. Procedia Eng..

[93] Dillner U., Kessler E., Meyer H.G. (2013). Figures of merit of thermoelectric and bolometric thermal radiation sensors. J. Sens. Sens. Syst..

[94] De Luca A., Cole M.T., Hopper R.H., Boual S., Warner J.H., Robertson A.R., Ali S.Z., Udrea F., Gardner J.W., Milne W.I. (2015). Enhanced spectroscopic gas sensors using in-situ grown carbon nanotubes. Appl. Phys. Lett..

[95] Luca A.D., Cole M.T., Fasoli A., Ali S.Z., Udrea F., Milne W.I. (2013). Enhanced infra-red emission from sub-millimeter microelectromechanical systems micro hotplates via inkjet deposited carbon nanoparticles and fullerenes. J. Appl. Phys..

[96] Ayerden N.P., Wolffenbuttel R.F. (2017). The Miniaturization of an Optical Absorption Spectrometer for Smart Sensing of Natural Gas. IEEE Trans. Ind. Electron..

[97] Podmore H., Scott A., Cheben P., Velasco A.V., Schmid J.H., Vachon M., Lee R. (2017). Demonstration of a compressive-sensing Fourier-transform on-chip spectrometer. Opt. Lett..

[98] Stark T., Imboden M., Kaya S., Mertiri A., Chang J., Erramilli S., Bishop D. (2016). MEMS Tunable Mid-Infrared Plasmonic Spectrometer. ACS Photonics.

[99] Mazen E., Yasser M.S., Mohammad S., Bassem M., Mostafa M., Diaa K. (2016). On-Chip Micro– Electro–Mechanical System Fourier Transform Infrared (MEMS FT-IR) Spectrometer-Based Gas Sensing. Appl. Spectrosc..

[100] Hopper R., Ali S.Z., Boual S., Luca A.D., Dai Y., Popa D., Udrea F. (2018). A CMOS-Based Thermopile Array Fabricated on a Single SiO_2_ Membrane. Proceedings.

[101] Varpula A., Timofeev A.V., Shchepetov A., Grigoras K., Hassel J., Ahopelto J., Ylilammi M., Prunnila M. (2017). Thermoelectric thermal detectors based on ultra-thin heavily doped single-crystal silicon membranes. Appl. Phys. Lett..

[102] Inoue T., Zoysa M.D., Asano T., Noda S. (2014). Realization of dynamic thermal emission control. Nat. Mater..

[103] Sollradl T., Ranacher C., Consani C., Puhringer G., Lodha S., Jakoby B., Grille T. Characterisation of a resonant-cavity enhanced thermal emitter for the mid-infrared. Proceedings of the 2017 IEEE SENSORS.

[104] Chen K., Adato R., Altug H. (2012). Dual-Band Perfect Absorber for Multispectral Plasmon-Enhanced Infrared Spectroscopy. ACS Nano.

[105] Liu N., Mesch M., Weiss T., Hentschel M., Giessen H. (2010). Infrared Perfect Absorber and Its Application As Plasmonic Sensor. Nano Lett..

[106] Tan X., Li J., Yang A., Liu H., Yi F. Narrowband plasmonic metamaterial absorber integrated pyroelectric detectors towards infrared gas sensing. Proceedings of the SPIE, Smart Photonic and Optoelectronic Integrated Circuits XX.

[107] Ghaderi M., Shahmarvandi E.K., Wolffenbuttel R.F. (2018). CMOS-compatible mid-IR metamaterial absorbers for out-of-band suppression in optical MEMS. Opt. Mater. Express.

[108] Suen J.Y., Fan K., Montoya J., Bingham C., Stenger V., Sriram S., Padilla W.J. (2017). Multifunctional metamaterial pyroelectric infrared detectors. Optica.

[109] Köhring M., Böttger S., Willer U., Schade W. (2015). LED-Absorption-QEPAS Sensor for Biogas Plants. Sensors.

[110] Malcovati P., Baschirotto A. (2018). The Evolution of Integrated Interfaces for MEMS Microphones. Micromachines.

[111] Fohrmann L.S., Sommer G., Pitruzzello G., Krauss T.F., Petrov A.Y., Eich M. 2D integrating cell waveguide platform employing ultra-long optical path lengths. Proceedings of the 2017 IEEE 14th International Conference on Group IV Photonics (GFP).

[112] Gervais A., Jean P., Shi W., LaRochelle S. (2019). Design of Slow-Light Subwavelength Grating Waveguides for Enhanced On-Chip Methane Sensing by Absorption Spectroscopy. IEEE J. Sel. Top. Quantum Electron..

